# Differential expression profiles of the salivary proteins SP15 and SP44 from *Phlebotomus papatasi*

**DOI:** 10.1186/s13071-016-1633-z

**Published:** 2016-06-24

**Authors:** Nasibeh Hosseini-Vasoukolaei, Farah Idali, Ali Khamesipour, Mohammad Reza Yaghoobi-Ershadi, Shaden Kamhawi, Jesus G. Valenzuela, Haleh Edalatkhah, Mohammad Hossein Arandian, Hossein Mirhendi, Shaghayegh Emami, Reza Jafari, Zahra Saeidi, Mahmood Jeddi-Tehrani, Amir Ahmad Akhavan

**Affiliations:** Department of Medical Entomology and Vector Control, School of Public Health, Tehran University of Medical Sciences, Tehran, Iran; Department of Medical Entomology and Vector Control, Health Sciences Research Center, Faculty of Health, Mazandaran University of Medical Sciences, Sari, Iran; Reproductive Immunology Research Center, Avicenna Research Institute, ACECR, Tehran, Iran; Center for Research and Training in Skin Diseases and Leprosy, Tehran University of Medical Sciences, Tehran, Iran; Vector Molecular Biology Section, Laboratory of Malaria and Vector Research, National Institute of Allergy and Infectious Diseases, National Institute of Health, Rockville, MD 20852 USA; Reproductive Biotechnology Research Center, Avicenna Research Institute, ACECR, Tehran, Iran; Esfahan Health Research Station, National Institute of Health Research, Tehran University of Medical Sciences, Esfahan, Iran; Department of Medical Mycology and Parasitology, School of Medicine, Isfahan University of Medical Sciences, Isfahan, Iran; Monoclonal Antibody Research Center, Avicenna Research Institute, ACECR, Tehran, Iran

**Keywords:** Salivary gene expression, *Phlebotomus papatasi*, PpSP15, PpSP44, Iran

## Abstract

**Background:**

Sand fly saliva has been shown to help parasite establishment and to induce immune responses in vertebrate hosts. In the current study, we investigated the pattern of expression of two *Phlebotomus papatasi* salivary transcripts in specific physiological and seasonal conditions at a hyperendemic area of zoonotic cutaneous leishmaniasis (ZCL) in Iran.

**Methods:**

Sand flies were collected during 2012–2013, and grouped according to physiological stages such as unfed, fed, semi-gravid, gravid, parous, nulliparous, infected or non-infected with *Leishmania major* and also based on the season in which they were collected. Quantitative Real-Time PCR was applied for assessment of the expression of two relevant salivary transcripts, PpSP15 and PpSP44, associated to protection from and exacerbation of ZCL, respectively.

**Results:**

The expression of PpSP15 and PpSP44 transcripts was significantly up-regulated (1.74 and 1.4 folds, respectively) in blood fed compared to unfed flies. Among four groups of fed, unfed, semi-gravid and gravid flies, the lowest levels of PpSP15 and PpSP44 expression were observed in gravid flies. Additionally, the expression levels of both PpSP15 and PpSP44 transcripts in *P. papatasi* collected during summer were significantly up-regulated (3.7 and 4.4 folds, respectively) compared to spring collections. In addition, the PpSP15 transcript exhibited a significant up-regulation (*P* < 0.05) in non-infected flies compared to those infected with *L. major*.

**Conclusions:**

This study contributes to our knowledge of the differential expression of salivary genes among different groups within a *P. papatasi* population under natural field conditions. Cutaneous and visceral leishmaniasis are of public health importance in many parts of Iran and neighbouring countries where *P. papatasi* is the proven and dominant sand fly vector for ZCL, the most prevalent and endemic form of the disease in Iran. Therefore, the current study could be helpful in understanding the influence of salivary genes on *Leishmania* transmission by phlebotomine sand flies. Our findings demonstrate the differential expression of salivary transcripts under various physiological conditions potentially influencing the sand fly capacity for parasite transmission as well as the outcome of disease.

**Electronic supplementary material:**

The online version of this article (doi:10.1186/s13071-016-1633-z) contains supplementary material, which is available to authorized users.

## Background

Zoonotic cutaneous leishmaniasis (ZCL) is a neglected tropical disease caused by the protozoan parasite *Leishmania major* [1]. ZCL is endemic in 17 out of 31 provinces of Iran and represents a public health problem of increasing proportions [2]. The incidence rate of ZCL in Esfahan Province, a hyperendemic zone of ZCL in central Iran, is reported to be around 2400 cases per year (communication from the Esfahan Center for Public Health). This is considered to be an underestimation of the actual incidence.

A sand fly salivates as it bites the vertebrate host skin. Salivary glands have a unicellular epithelial layer surrounding a container for saliva consisting of a repertoire of proteins that vary based upon the physiological state of adults, sex, age, generation, species and geographical location of the sand fly [3, 4]. Sand fly saliva contains a series of bioactive molecules which are necessary for the successful uptake of blood meals and for establishment of *Leishmania* in vertebrate hosts [5, 6].

Sand fly saliva has immunomodulatory characteristics and induces a specific immunity consisting of antibody production and a cellular immune response [7]. Recently, the biological activity and immunogenicity of sand fly salivary proteins were comprehensively reviewed [8]. A protein called PpSP15 from *P. papatasi* saliva is a member of the small odorant binding-like family of proteins [9]. The homologue of this protein in *P. duboscqi*, PdSP15, was recently shown to inhibit contact pathway activation by binding to negatively charged molecules including polyphosphate, heparin, and dextran Sulfate [10]. PpSP44, another protein from *P. papatasi* saliva is a member of the yellow family of proteins found in all sand fly species [9]. The function of the sand fly yellow family of proteins was recently characterized as proteins that bind bioamines including epinephrine and norepinephrine [11]. Both proteins are also immunogenic. PpSP15 protein from *P. papatasi* saliva induced an immunity in rodents that conferred protection against *Leishmania major* infection [12, 13]. The observed protection correlated with the induction of a specific delayed-type hypersensitivity response (DTH) with a Th1 profile [12]. These studies introduced PpSP15 as a candidate vaccine against infection with *L. major*. Interestingly, immunization with PpSP44 produced a Th2 response in mice that enhanced *L. major* infection [13]. This study demonstrated a differential immune response to distinct molecules in saliva of the same sand fly species leading to different outcomes of the disease [13].

Biologically active molecules in sand fly saliva are conserved for some proteins and divergent for others [14–18]. Few studies have been aimed at understanding the effect of physiological and seasonal factors on the expression of sand fly salivary proteins and their subsequent effect on parasite transmission and epidemiology of leishmaniasis.

*Phlebotomus papatasi* is the main vector of ZCL in the Old World and Iran [19, 20]. The behaviour of this sand fly species is well documented with regards to resting places [21], blood sources [22–25], bacterial microflora [26], longevity [27], dispersal ability [28] and seasonal activity [29, 30]. Moreover, many studies have demonstrated a role for biotic or abiotic factors on gene expression [31]. Although the physiology and ecology of *P. papatasi* sand flies are well known, the effect of the physiological state and the environment on salivary gene expression in this insect is still unknown.

In this study we tested the hypothesis that the expression profiles of PpSP15 and PpSP44 salivary transcripts varies with the physiological state and seasonal status of female *P. papatasi* sand flies, and that these variations are different in the two salivary transcripts potentially impacting the host immune response.

## Methods

### Study area

**Fig. 1:**
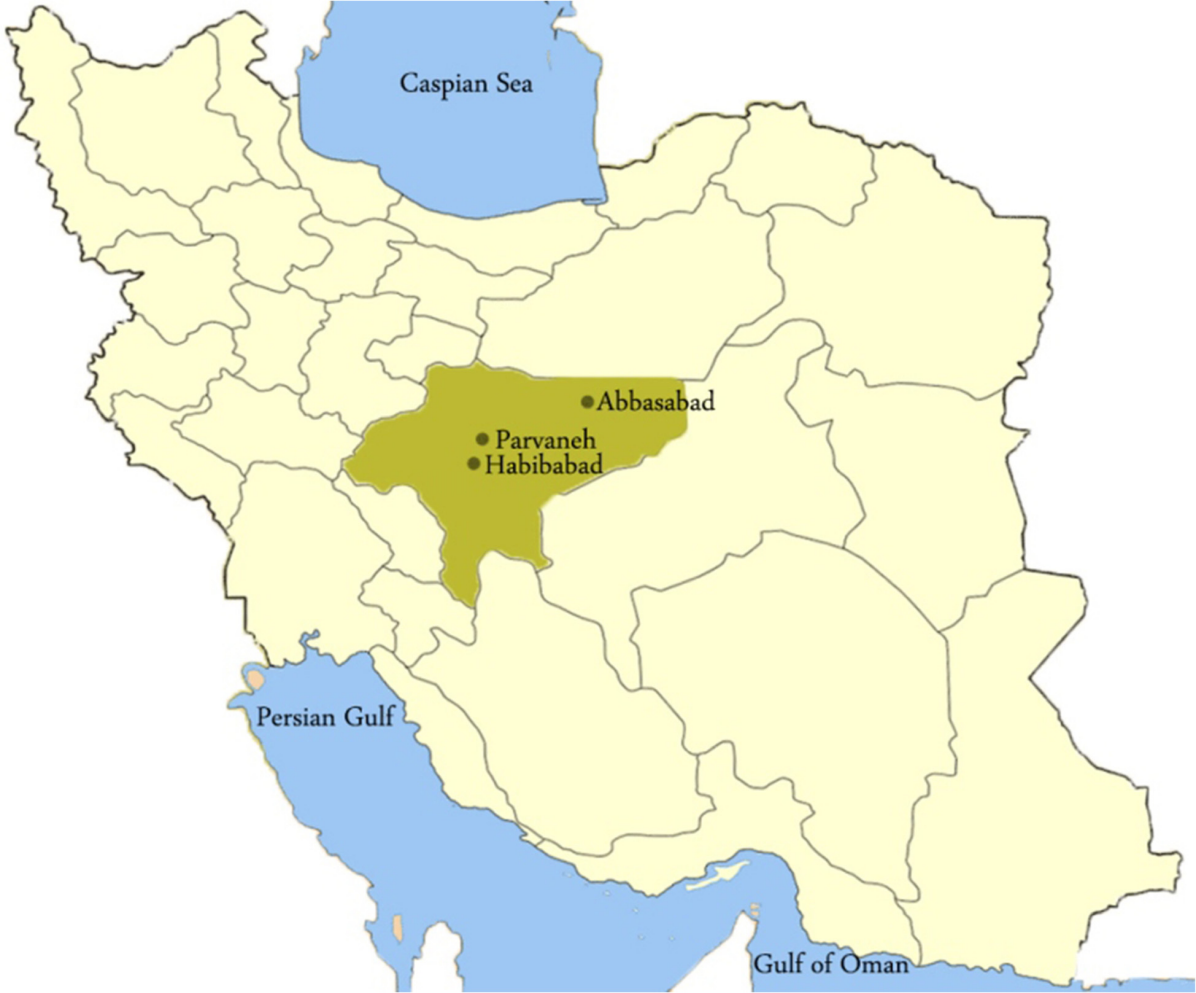
Geographical location of Abbasabad, Habibabad and Parvaneh-Aliabadchi villages in Esfahan Province, central Iran

This investigation was accomplished during 2012–2013 in three villages of Parvaneh-Aliabadchi, Habib Abad and Abbasabad in Esfahan province, central Iran (Fig. 1). The Habib Abad and Parvaneh- Aliabadchi villages are located 25–40 km north of the city of Esfahan (32°39′35"N, 51°40′17"E). The Abbasabad village is located 5 Km from Badroud district (33°42′N, 52°2′E), Natanz city, Esfahan province, central Iran.

The biotope of the selected areas is desert with hot summers and cold winters. The two study areas near Esfahan city are located at an altitude of around 1550 m, and Badroud district is located at an altitude of 1056 m, in the foothills of Karkas Mountains. Wheat, barley, cotton, vines, beetroot, pistachio, alfalfa, Indian corn, clover and summer crops are cultivated in these areas (the Esfahan Agriculture Organization).

In 2013, the maximum and minimum monthly temperatures in Esfahan city were 39.8 °C in July and -5.3 °C in December. The maximum and minimum monthly relative humidity were 81 % and 9.1 % in November and July, respectively. In Badrood district, the maximum and minimum monthly temperatures were 43 °C in July and -2.9 °C in December, respectively. The maximum relative humidity was 62 % in December and the minimum was 20 % in February. The total annual rainfall was 84.6 mm in Esfahan city and 77.5 mm in Badrood district (Esfahan Metrological Organization).

### Sand fly collection and rearing

Phlebotomines were collected using aspirating tubes and funnel trapping from resting places throughout the selected study villages during the active season of sand flies in 2012–2013. Collected sand flies were moved into cloth cages with 20 × 20 × 20 cm dimensions, hanging on steel frames and then transferred to the insectary of Esfahan Training and Health Research Center (ETHRC), National Institute of Health Research (NIHR), Tehran University of Medical Sciences (TUMS), Esfahan, Iran. Sand flies were maintained at ETHRC on a 14:10 LD photoperiod, at 26–28 °C, 80 % relative humidity and adult sand flies were fed on a 20 % solution of sucrose on cotton wool.

Sand flies were identified according to morphological characters using a systematic key [32]. Female *P. papatasi* were separated from other species for inclusion in the study and categorized into ten groups according to the following biological and environmental factors: accessory gland status, parous and nulliparous and physiological status of unfed, fed, semi-gravid and gravid. Two groups of sand flies were collected throughout spring and summer. According to their infection status two groups of *L. major*-infected and non-infected were also categorized (Table 1). For detection and identification of *Leishmania* species in *P. papatasi*, nested PCRs were done using *Leishmania* ITS2 specific primers [33]. Salivary gene expression profiles of parous versus nulliparous, *L. major*-infected versus non-infected, spring versus summer collection and unfed versus fed, semi-gravid and gravid were compared.

**Table 1:** Grouping of collected *Phlebotomus papatasi* according to some physiological and environmental features

Features	Accessory glands status	Physiological stages	Seasons	Infection with *L. major*
Groups	1-Parous	1-Unfed	1-Spring	1-Infected
	2-Nulliparous	2-Fed	2-Summer	2-Non-infected
		3-Semi-gravid		
		4-Gravid		

### Salivary gland preservation in RNAlater

Salivary glands of collected *P. papatasi* females were dissected and the head along with the attached salivary glands was carefully separated from the body and preserved in 1.5 ml microtubes containing RNAlater® solution (Qiagen, Germany) and incubated at 4 °C overnight then stored at -20 °C until use.

### Expression of salivary gland genes of *Phlebotomus papatasi*

In this study the expression pattern of salivary gland genes of *P. papatasi* was determined in ten different groups of sand fly as described above (Table 1). Two salivary genes of *P. papatasi* namely PpSP15 and PpSP44 were studied in this project. The amount of PpSP15 and PpSP44 transcript expression was determined by quantitative Real-Time PCR (qRT-PCR). Total RNA was isolated from salivary tissues followed by complementary DNA (cDNA) synthesis that was later used as a template for qRT PCR.

### RNA isolation

Head plus salivary glands, preserved in RNAlater, were removed using sterile forceps and submerged in RNAzole® RT (MRC, OH, USA) for total RNA isolation. Because of the small size of an individual sand fly, total RNA was isolated from a pool of ten sand fly heads plus salivary glands from each group.

### DNase I treatment

RNA samples isolated from heads plus salivary glands of *P. papatasi* were treated with DNase I enzyme to avoid any possible genomic DNA contamination. For removal of genomic DNA the following procedure was carried out: 1 μl (1 u) DNase I, RNase free enzyme (Fermentas, UK), 1 μl 10X reaction buffer with MgCl_2_, 1 μg RNA, RiboLock™ RNase inhibitor enzyme (1u/μl) and DEPC-treated water were added to a final volume of 10 μl and incubated at 37 °C for 30 min. Then 1 μl 50 mM EDTA was added and incubated at 65 °C for 10 min.

To assess the quality and quantity of the isolated RNA, 3 μl RNA was run on 1 % agarose gel and visualized using ethidium bromide. The density of extracted RNA was measured by Picodrop microliter UV/Vis spectrophotometer (Picodrop, Cambridge, UK). Isolated RNA samples containing at least 1 μg RNA were included in the study; samples without enough RNA were excluded.

### cDNA synthesis

We used 1 μg of total RNA per 20 μl cDNA reaction. For cDNA synthesis, 10 μl of RNA were incubated at 65 °C for 10 min. Then the following reagents were added to the RNA templates to a final volume of 20 μl: Four μl of 5X reaction buffer, 1 μl (200 u) Revert Aid™ reverse transcriptase (Fermentas, UK), 1 μl random hexamer primer (20 pmol), 0.5 μl (20 u) RNasin® Plus RNase inhibitor (Promega, USA), 2 μl dNTP mix (10 mM) and 1.5 μl DEPC-treated water. The sample tubes were placed in a thermo cycler (Eppendorf, Hamburg, Germany) and run using the following programme: 25 °C for 10 min, 42 °C for 1 h and 72 °C for 5 min.

### Quantitative Real-Time polymerase chain reaction

Real-Time PCRs were performed using Maxima SYBR green and Rotor-Gene Q instrument (Qiagen, Germany). The qRT-PCR reactions were performed in duplicates using 6.25 μl SYBR green master mix (Fermentas, UK), 1 μl (0.8 μM) each of forward and reverse primers, 1 μl cDNA and 3.25 μl DNase/RNase-Free water to a final volume of 12.5 μl in 0.1 ml capillary tubes. Samples were then put in Rotor-Gene Q and the qRT-PCR reaction initiated with a step at 95 °C for 10 min, followed by 40 cycles of 95 °C for 15 s, 60 °C for 30s and 72 °C for 30s.

Expression of PpSP15 and PpSP44 salivary gland genes were assessed in each of ten sand fly groups. Each reaction was repeated four times for each gene (in duplicate in two different runs). Specific primers for SP15 F, SP15 R, SP44 F and SP44 R were used for the salivary genes, and TUB F and TUB R for the alpha-tubulin housekeeping control [34].

### Data analysis

The data obtained from qRT-PCR were analyzed using the relative standard curve method to determine n-fold differences of PpSP15 and PpSP44 salivary gene expression relative to the calibrator in different groups of *P. papatasi*. In our experiments salivary glands from newly emergent unfed nulliparous laboratory-reared female sand flies were used as calibrators.

### Calculation steps of the relative standard curve method

Step 1: Normalization to endogenous control:

$$ \mathrm{Normalized}\ \mathrm{concentration} = \frac{\mathrm{Concentration}\ \mathrm{of}\ \mathrm{specific}\ \mathrm{gene}}{\mathrm{Concentration}\ \mathrm{of}\ \mathrm{alpha}\ \mathrm{tabulin}\ \mathrm{gene}} $$

Step 2: Normalization to calibrator sample:

$$ \mathrm{Fold}\ \mathrm{difference} = \frac{\mathrm{Normalized}\ \mathrm{concentration}\ \mathrm{of}\ \mathrm{sample}}{\mathrm{Normalized}\ \mathrm{concentration}\ \mathrm{of}\ \mathrm{calibrator}} $$

In our study standard curves were run in all qRT-PCR reactions for more accurate quantitative results. Both the target genes and the housekeeping alpha tubulin gene were simultaneously examined in each run of qRT-PCR. Large pools of calibrator cDNA were generated and aliquoted into single-use tubes. Standard curves were generated using calibrator sample pools and the same calibrator pools were used in all reactions throughout the study to achieve constant real time PCR results.

### Statistical analysis

Statistical analyses were performed using GraphPad Prism v.5.04 (Graphpad software Inc., San Diego, CA, USA). The nonparametric Kruskal-Wallis statistical test was used for comparison among data sets of more than two groups and if the test was statistically significant, the nonparametric Mann-Whitney *U* test was used for comparison between data sets of two groups. Correlation between the expression profiles of two salivary genes was determined using the Spearman correlation test. *P- * values less than 0.05 were considered as significant.

## Results

The expression profiles of PpSP15 and PpSP44 salivary genes were determined for female *P. papatasi* sand flies at different physiological stages and in different environmental factors. Overall, the expression of PpSP15 and PpSP44 transcripts was regulated by physiological and environmental factors such as blood meal, season, parity and parasite infection. Fold change ratios ranged from 0.51 to 4.9 for PpSP15 and 0.38 to 5.2 for PpSP44 when the effects of physiological and environmental factors on salivary gene expression were analyzed (*df *= 3, *χ*^2^ = 14.12, *P* = 0.0027 and *χ*^2 ^= 13.79, *P* = 0.0032, for PpSP15 and PpSP44, respectively) (Table 2).

**Table 2:** Relative expression of PpSP15 and PpSP44 in different groups of *Phlebotumus papatasi*

Sand fly groups	PpSP15	PpSP44	Sand fly groups	PpSP15	PpSP44
	Fold difference	Fold difference		Fold difference	Fold difference
Fed	4.33	5.16	Unfed	2.31	3.79
	3.90	4.71		2.21	3.66
	4.87	4.25		2.57	3.18
	4.66	4.19		2.54	2.69
Mean ± SEM	4.44 ± 0.21	4.58 ± 0.23	Mean ± SEM	2.41 ± 0.09	3.33 ± 0.25
Semi-gravid	2.67	2.50	Gravid	1.22	1.30
	2.93	2.64		1.15	1.23
	2.62	2.57		1.21	1.25
	2.73	2.73		1.12	1.24
Mean ± SEM	2.74 ± 0.07	2.60 ± 0.05	Mean ± SEM	1.17 ± 0.02	1.26 ± 0.02
Spring	0.51	0.41	Summer	2.14	2.14
	0.52	0.38		1.92	1.83
	0.71	0.57		2.46	2.30
	0.59	0.51		2.11	2.02
Mean ± SEM	0.58 ± 0.05	0.47 ± 0.04	Mean ± SEM	2.16 ± 0.11	2.07 ± 0.10
Parous	2.39	1.24	Nulliparous	1.78	1.15
	2.20	1.29		1.93	1.25
	2.84	1.49		2.18	1.36
	2.41	1.14		2.28	1.65
Mean ± SEM	2.46 ± 0.14	1.29 ± 0.07	Mean ± SEM	2.04 ± 0.12	1.35 ± 0.11
Infected	1.46	0.99	Non-infected	1.58	0.99
	1.33	0.83		1.71	0.99
	1.50	0.93		1.57	0.85
	1.44	0.87		1.90	0.97
Mean ± SEM	1.43 ± 0.03	0.90 ± 0.03	Mean ± SEM	1.69 ± 0.08	0.95 ± 0.03

**Fig. 2:**
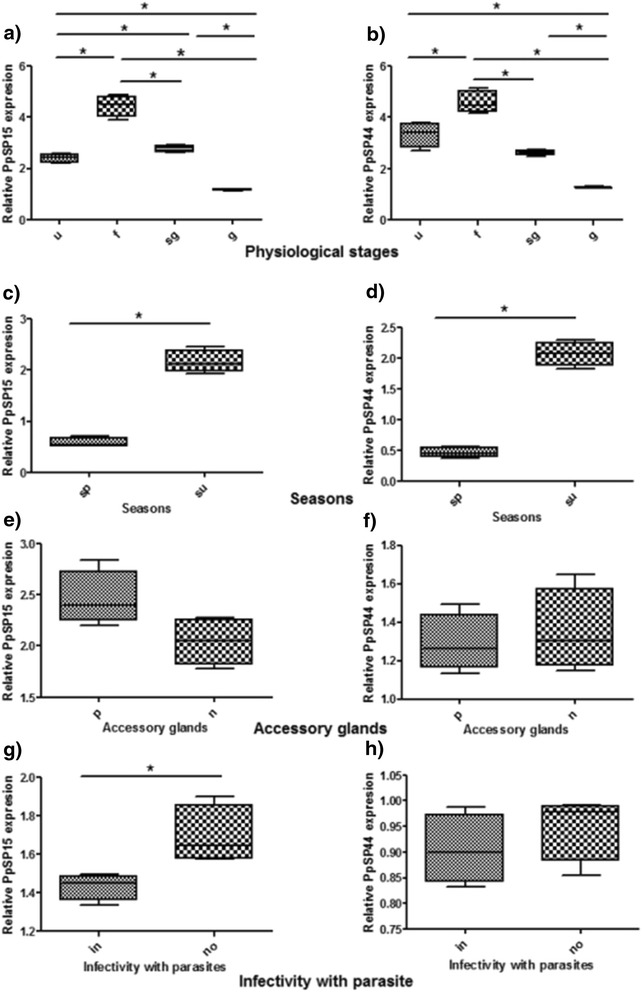
The relative expression of PpSP15 and PpSP44 salivary transcripts in different *Phlebotomus papatasi* groups. **a**, **b**) physiological stages: u (unfed), f (fed), sg (semi-gravid), g (gravid), **c**, **d**) seasons: sp (spring), su (summer), **e**, **f**) accessory glands: p (parous), n (nulliparous), **g**, **h**) infectivity with parasite: in (infected) and no (non-infected), *P < 0.05

Testing the effect of blood meal on expression of PpSP15 in collected *P. papatasi* showed 2.41 ± 0.09 fold change in unfed vs. 4.44 ± 0.21 in fed group (*P* = 0.0286). Semi-gravid flies expressed a significantly higher level of the PpSP15 transcript (2.74 ± 0.07) than gravid flies (1.17 ± 0.02) (*P* = 0.0286). Fed sand flies showed a significant 1.8 fold higher PpSP15 gene expression than unfed ones (*P* = 0.0286), 1.6 fold higher than semi-gravid (*P* = 0.0286) and 3.8 fold higher than gravid (*P* = 0.0286) sand flies (Table 2). Unfed and semi-gravid sand flies expressed significant 2.06 and 2.34 fold higher PpSP15 expression than gravid sand flies, respectively (*P* = 0.0286). The significant difference in PpSP15 gene expression was also observed between unfed and semi-gravid sand flies. The expression profiles were fed > semi-gravid > unfed > gravid (Fig. 2a).

The transcript level of PpSP44 showed a 3.33 ± 0.25 fold increase in unfed *vs* 4.58 ± 0.23 in the fed group (*P* = 0.0286). Semi-gravid flies expressed a significantly higher level of the PpSP44 transcript (2.6 ± 0.05) than gravid flies (1.26 ± 0.02) (*P* = 0.0286) (Table 2). In this regard, the gene expression level of PpSP44 was highest in fed and lowest in gravid sand flies. The expression profile of PpSP44 among these four groups was: fed > unfed > semi-gravid > gravid (Fig. 2b). Fed sand flies showed a significant 1.4 fold higher PpSP44 gene expression than unfed (*P* = 0.0286), 1.8 fold higher than semi-gravid (*P* = 0.0286) and 3.6 fold higher than gravid (*P* = 0.0286) sand flies. Gravid sand flies showed a significant 2.6 fold lower PpSP44 expression than unfed (*P* = 0.0286) and 2.1 fold lower than semi-gravid (*P* = 0.0286) sand flies (Table 2, Fig. 2b).

The summer collected sand flies showed fold changes of 2.16 ± 0.11 and 2.07 ± 0.10 compared to the spring collection with fold changes of 0.58 ± 0.05 and 0.47 ± 0.04 for PpSP15 and PpSP44, respectively (Table 2). The level of PpSP15 and PpSP44 gene expression in sand flies collected during summer was a significant 3.7 and 4.4 fold higher than those sand flies collected during spring, respectively (*P* = 0.0286) (Fig. 2c, d).

The fold change for parous and nulliparous groups of sand flies, respectively, were 2.46 ± 0.14 and 2.04 ± 0.12 for PpSP15 expression and 1.29 ± 0.07 and 1.35 ± 0.11 fold for PpSP44 expression (Table 2). The expression level of PpSP15 gene in parous sand flies was higher than in nulliparous sand flies but its expression levels and that of PpSP44 did not differ significantly between these two groups (Fig. 2e, f).

Gene expression in infected and non-infected sand flies, respectively, showed a 1.43 ± 0.03 and 1.69 ± 0.08 fold change for PpSP15 and 0.9 ± 0.03 and 0.95 ± 0.03 fold change for PpSP44 (Table 2). In *L. major*-infected *P. papatasi* sand flies the PpSP15 gene expression was a significant 1.2 fold lower (*P* = 0.0286) than in non infected ones (Fig. 2g, h).

**Fig. 3:**
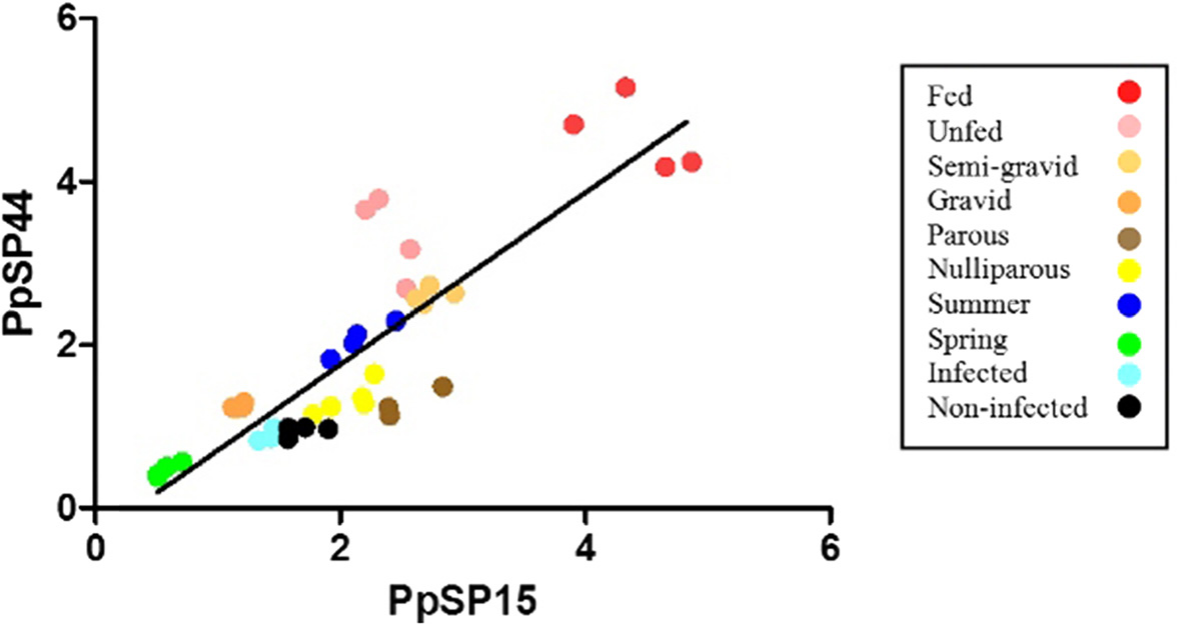
Correlation between PpSP15 and PpSP44 relative transcript expression in different sand fly groups. r: 0.8460, *P* < 0.0001

The correlation between expression profiles of PpSP15 and PpSP44 genes was determined using the Spearman correlation test. There was a strong positive correlation between the expression of these two salivary genes, in 10 sand fly groups (*r*_(__PpSP15,PpSP44__) _= 0.85, *P* < 0.0001) (Fig. 3).

## Discussion

Genetic variations in sand fly saliva influence the process of developing a vaccine against leishmaniasis. The study of Elnaiem et al. [15] suggested that the high degree of similarity in *P. papatasi* SP15 between different populations may be used in a vaccination strategy. The authors concluded that no major variation at the genetic and amino acid level of PpSP15 reinforces its suitability as a vaccine candidate. In contrast, several studies on Maxadilan, a salivary protein in new world *Lutzomyia longipalpis*, demonstrated that this protein has a high amount of genetic and amino acid variations and consequently the host immune response to each variant is specific, therefore reducing its value as a potential vaccine candidate [35–37]. In addition to genetic variability, differences in the level of salivary gene expression could also influence vaccine development.

In this study we addressed the hypothesis that some physiological aspects such as blood feeding, parity or nulliparity, as well as *Leishmania* infection and seasonal status modulate the expression profiles of two of the most significant transcripts found in the salivary glands of female *P. papatasi* sand flies [12, 13].

In four groups of blood fed, unfed, semi-gravid and gravid flies the expression levels of salivary transcripts of PpSP15 and PpSP44 genes were assessed. Supporting the hypothesis, expression of the two genes was up-regulated in blood fed compared to unfed flies. In accordance with our results, a recent study demonstrated the up-regulation of PpSP12, PpSP14, PpSP15, PpSP30, PpSP36, PpSP42 and PpSP44 salivary gland gene expression in blood fed laboratory reared *P. papatasi* [34]. This induction of salivary transcripts following blood feeding of sand flies reflects the important role of salivation during feeding. Higher gene expression in fed flies may be because of the subsequent need for regeneration of salivary proteins after a meal in preparation for the next blood feeding. In accordance with this finding, previous studies showed that the total amount of salivary proteins reduces following a blood meal [38–40]. In another study in our laboratory, the lower levels of salivary proteins in fed *P. papatasi* flies was also in agreement with this finding [41]. Lower mRNA expression in unfed flies may be an indication that sand flies have stored enough saliva in their glands that could be used during the feeding process, down regulating salivary gene transcription.

Among four groups of fed, unfed, semi-gravid and gravid flies, the lowest levels of PpSP15 and PpSP44 gene transcripts were observed in gravid sand flies. Although the physiological processes that may be in charge of such differences in salivary gland gene expressions between fed and gravid flies or between semi-gravid and gravid flies are not known, these findings should be considered in future examinations of the interaction between the salivary gene expression and sand fly physiological status.

Aging is another physiological factor we hypothesized may influence gene expression profiles, however, it is not thought to be as significant as genotype or sex in insects [42]. As nulliparous flies have never oviposited, parity is usually used to determine the age structure of a population. In the present study, only PpSP15 appeared to be influenced by parity. In contrast, the modulation of PpSP44 expression profile was not statistically significant between parous and nulliparous flies. A higher expression level of PpSP15 was observed in parous flies which are older compared to nulliparous flies. In accordance with our finding, the expression level of PpSP15 genes was greater in 9 day blood-fed *P. papatasi* compared to 5 day flies [34]. The results of the current study show that there is a direct relationship between the up-regulation of the PpSP15 transcript and aging. In a previous study conducted in the Esfahan province, a ZCL hyperendemic area of Iran, most of the detected *Leishmania* parasites were from parous compared to nulliparous *P. papatasi* sand flies [30]. It remains to be determined whether the observed up-regulation of PpSP15 in older flies modulates the host immune response following parasite transmission.

The activity of sand flies in central parts of Iran with temperate climates, starts from April or May and extends to October or November with two peaks of activities, one in June or July and the other in August or September [29, 30]. The expression levels of both PpSP15 and PpSP44 salivary transcripts were up-regulated in female *P. papatasi* collected during the summer as compared to spring collected flies. In the current study we have three hypotheses associated with this up-regulation: a) Changes in relative temperature and humidity between seasons influence vegetation and the habitat of sand flies causing differential expression of salivary genes; b) As a population, sand flies in the summer are older than in the spring and therefore their salivary repertoire may have matured. c) Up-regulation may have an association with the increased rate of disease transmission in summer. In an epidemiological survey of ZCL in Esfahan Province, *Leishmania* infection was detected in *P. papatasi* from mid-May until the end of October and the greatest infection rate of *P. papatasi* was in September [25]. This correlated to the highest rate of leishmanial infections in *R. opimus* from Esfahan province, also observed in September, while the lowest rate was reported in April [43]. In another survey in this area the highest prevalence of CL was observed in autumn and the lowest in spring [44]. In Jordan, the increased expression of PpSP44 in *P. papatasi* collected in September, late in the sand fly season, was attributed to a dryer environment and the scarcity of sugar sources [45].

Importantly, PpSP15 exhibited a significant up-regulation in non-infected flies compared to those infected with *L. major*. Though not directly demonstrated, it may be possible that saliva is modulated by *Leishmania* parasites to promote its transmission, causing sand flies to probe more and therefore provide a greater chance for parasites to be injected in the skin. Indeed, modulation of salivary protein expression by trypanosomes and *Plasmodium* has been reported in tse tse flies and *Anopheles* mosquitoes, respectively [46, 47].

Of note, the expression profiles of PpSP15 and PpSP44 were not contrasting but positively correlated suggesting that these two salivary transcripts might follow a similar regulation pattern in the different sand fly states. It is important to determine whether a similar regulation of expression is observed for other salivary genes.

## Conclusion

In summary, the results of this study demonstrated the differential expression of two salivary transcripts in different physiological states within a *P. papatasi* population under natural field conditions. The observed influence of physiological and seasonal parameters on the composition of *P. papatasi* saliva needs further assessment to investigate their potential impact on parasite transmission and disease establishment.

## Abbreviations

cDNA, complementary deoxyribonucleic acid; DTH, delayed-type hypersensitivity; ETHRC, Esfahan Training and Health Research Center; f, fed; g, gravid; in, infected; n, nulliparous; NIHR, National Institute of Health Research; no, non infected; p, parous; PpSP15, *Phlebotomus papatasi* SP15; PpSP44, *Phlebotomus papatasi* SP44; qRT-PCR, quantitative real-time polymerase chain reaction; sg, semi-gravid; sp, spring; su, summer; Th1, type 1 helper T cell; TUMS, Tehran University of Medical Sciences; u, unfed; ZCL, zoonotic cutaneous leishmaniasis.

## Additional file

Additional file 1: Sequences of primers used in qRT-PCR. (XLS 24 kb)
